# Body Mass Index and Melanoma Prognosis

**DOI:** 10.5826/dpc.1104a106

**Published:** 2021-10-01

**Authors:** Nicoletta Cassano, Stefano Caccavale, Gino A. Vena, Giuseppe Argenziano

**Affiliations:** 1 Dermatology and Venereology Private Practice, Bari, Italy; 2 Dermatology and Venereology Private Practice, Barletta, Italy; 3 Dermatology Unit, University of Campania Luigi Vanvitelli, Naples, Italy

**Keywords:** cutaneous melanoma, body mass index, Breslow thickness, obesity

## Abstract

**Introduction:**

Obesity has been suggested as a risk factor in the progression of malignancies, including melanoma. Most studies defined obesity using body mass index (BMI), although the index is considered an imperfect measure of body composition.

**Objective:**

The aim of this article is to examine whether BMI can impact on the prognosis of cutaneous melanoma, regardless of anti-tumor therapy. The relationship between BMI and specific prognostic factors in melanoma patients has been reviewed.

**Methods:**

Literature search was conducted on PubMed using the terms “melanoma” and “body mass index” or “obesity”. We selected articles, published up to 30 November 2020, examining the prognostic aspects of melanoma. Articles evaluating the risk and incidence of melanoma were excluded as well as studies regarding morbidity and complications following surgical procedures, or those performed in metastatic melanoma patients treated with anti-tumor therapies.

**Results:**

Mixed results have emerged from studies assessing the clinical outcomes in melanoma patients in relation to BMI. More consistent data seem to support the relationship between BMI and Breslow thickness.

**Conclusions:**

Studies that focus specifically on the link between obesity and melanoma prognosis are limited; further research is needed to deepen our knowledge on this link.

## Introduction

There is a growing interest in exploring the relationship between cancer and anthropometric measures, including body mass index (BMI). Excess body weight, accounting for both overweight (BMI within the range of 25–29.9 kg/m^2^) and obesity (BMI≥30 kg/m^2^), has notably increased worldwide over the last decades and has been associated with a higher risk for cancer of several anatomic sites [1].

Obese status is characterized by the occurrence of systemic and tissue processes that might influence malignancies, such as release of cytokines and hormones from adipose tissues, chronic low-grade inflammation, increased estrogen levels, insulin resistance and hyperinsulinemia [2–4]. Obesity has also been suggested to contribute to the risk and progression of cutaneous melanoma, whose major environmental risk factor is ultraviolet radiation (UV), especially as intermittent intense exposures. However, the analysis of the association between obesity and risk of melanoma has provided conflicting data so far [4,5].

Recent studies have shown that increased BMI might improve outcomes in melanoma patients treated with targeted therapy and immunotherapy, providing further hints on the phenomenon known as the “obesity paradox”, although a lack of consistency has emerged from the currently available results and the issue is still under debate [6–9].

The aim of this article is to examine whether BMI can impact on the prognosis of cutaneous melanoma, regardless of anti-tumor therapy. For this purpose, the relationship between BMI and specific prognostic factors in melanoma patients has been reviewed.

## Methods

Articles in English published up to 30 November 2020 were obtained from the PubMed database. A literature search was conducted using the terms “melanoma” and “body mass index” or “obesity”. We collected full article copies that were considered potentially eligible, including review articles as appropriate. The reference lists of retrieved manuscripts were also checked to find other eligible papers. We selected articles focused on prognostic aspects of melanoma (e.g., mortality/survival, relapse, metastases, progression, and histologic prognostic factors). Articles investigating the risk and incidence of melanoma were excluded, as well as those on morbidity and complications after surgical procedures. Studies regarding patients with metastatic melanoma treated with anti-tumor therapies have also been excluded from our review process, as this topic was beyond the purposes of this manuscript and would deserve a separate wide discussion.

## Results

### Melanoma Outcomes

Only a limited number of studies have evaluated the survival rate and/or the risk of recurrence or progression in relation to BMI among melanoma patients. As previously specified, our analysis did not include studies assessing outcomes in patients with metastatic melanoma who received targeted therapy, immunotherapy, or chemotherapy.

No significant variations in the overall risk of mortality for melanoma according to BMI were detected in a prospective cohort study involving nearly 1.2 million UK women aged 50–64 years who were recruited into the Million Women Study during the period 1996–2001 and followed up, on average, for 5.4 years [10]. Similarly, a study on more than 900,000 adults in the USA showed no increased mortality rates from melanoma for higher categories of BMI in males or females [11].

In a total of 340 Italian melanoma patients (mean Breslow thickness=0.4 mm), prognosis was found to be similar in normal weight patients and in overweight/obese patients [12].

A recent analysis of the Leeds Melanoma Cohort, with a median follow-up length of 6.7 years, showed that BMI was not associated with overall and melanoma-specific survival [13].

The comparison of 131 relapsed melanoma patients with 147 non-relapsers reported no effect of BMI on the risk of relapse [14].

In a small study that found a correlation between serum levels of leptin and sentinel lymph node metastases in melanoma patients, the mean BMI was identical for the sentinel node-positive and sentinel node-negative groups, ruling out obesity as an explanation for higher leptin values in patients with positive sentinel nodes [15].

Instead, other findings seemed to indicate a variable influence of BMI on melanoma outcomes.

A study of the Leeds Melanoma Cohort, comprising 2,182 melanoma patients enrolled in the period 2001–2013, has revealed that BMI was not significantly associated with survival when it was treated as a continuous variable [hazard ratio (HR) 1.04 per 5 units, 95% confidence interval (CI) 0.91–1.18; P = 0.6)] [16]. Instead, overweight individuals had better survival than subjects with normal weight after adjustment for age and sex, and after further adjustment for site and Breslow thickness. The protective effect was not observed in obese patients. An analysis of data from participants in the same cohort previously showed that BMI was predictive of relapse even when corrected for Breslow thickness [17].

Increased BMI has been associated with worse survival in an USA investigation of 1,186 patients with surgically resected melanoma, 75% of whom had stage I or II disease [18]. Overweight patients showed a trend towards elevated risks of disease recurrence and death; such risks were significantly increased in obese patients (P < 0.05). However, outcome associations were weakened or lost their significance following adjustment for C-reactive protein (CRP).

In a retrospective analysis of 261 Korean patients with primary cutaneous melanoma, overweight and obesity (BMI>23 kg/m^2^) were significantly associated with the development of metastases [odds ratio (OR)=2.10, 95% CI 1.2–3.6] and with shorter overall survival (P = 0.033) [19]. It should be high-lighted that the cut-off values for the definition of overweight and obesity are lower for Asian populations as recommended by the World Health Organization (WHO), and in the Korean study overweight status was defined as a BMI of 23 to 24.9 kg/m^2^, while a BMI≥25 kg/m^2^ defined the obesity status [19,20].

### Breslow Thickness and Other Histological Prognostic Factors

Some studies have investigated the relationship between BMI and Breslow thickness. The characteristics of the largest and most important studies are summarized in Table 1.

De Giorgi et al reported no association between over-weight status (BMI≥25 kg/m^2^) and the risk of thick melanoma (Breslow thickness more than 1 mm) in the total sample or men, whereas a trend towards association between BMI≥25 kg/m^2^ and the risk of thick melanoma was shown among women (OR=1.64, 95% CI 0.82–3.28), and especially post-menopausal women (OR=2.50, 95% CI 1.06–5.88) [21].

In the report by Gandini et al [22], BMI was independently identified as significantly associated with Breslow thickness. In the multivariate random effects model, median Breslow thickness was 1.2 for BMI≥25 kg/m^2^ versus 0.8 for BMI<25 kg/m^2^ (P = 0.0008), and the multivariate logistic model disclosed a significant association of higher BMI with thick melanoma [the comparison of BMI≥25 kg/m^2^ vs. BMI<25 kg/m^2^ produced an OR of 1.34 (95% CI 1.12–1.59; P = 0.001)].

Skowron et al reported that BMI≥30 kg/m^2^ was associated with the risk of higher Breslow thickness (OR=2.78, 95% CI 1.55–4.94; P = 0.001) [23]. In the multivariate analysis of significant clinical and histological criteria, these authors found that obesity had an increased risk of higher tumor thickness (OR=1.86, 95% CI 0.91–3.77), but without any statistical significance (P = 0.086). When considering only clinical features in the model, obesity was significantly associated with a risk of higher Breslow thickness (OR=2.33, 95% CI 1.21–4.49; P = 0.011). The clinical characteristics of melanoma, its topography and visibility were not associated with the distribution of BMI categories. Instead, melanoma subtypes were differently distributed according to BMI categories (P = 0.007), with superficial spreading, lentigo maligna, and unclassified melanomas mostly found in patients with normal BMI, acral lentiginous melanoma in preobese patients and nodular melanoma in the obese patients [23].

Following these publications, the study of Stenehjem et al was the first to model Breslow thickness as a continuous outcome in relation to anthropometric measures which were obtained prediagnostically [24]. Breslow thickness was found to be significantly increased with increasing values of BMI (P_trend_ = 0.009). A BMI>30 kg/m^2^ was associated with significantly higher tumor thickness as compared to normal weight patients (geometric mean ratio 1.16, 95% CI 1.04–1.30). When melanomas were stratified by anatomical site and histological variants, significant positive trends were seen for continuous variables of BMI in trunk and lower limb melanomas and in superficial spreading melanomas, respectively, but not in melanomas localized in other sites or in other histological subtypes. The shape of the exposure–response curves indicated that mean Breslow thickness increased until a BMI of 29 kg/m^2^, then plateaued at a mean of approximately 2.5 mm before declining for the highest values. In addition, when Breslow thickness was examined as a dichotomous outcome according to BMI in overweight and postmenopausal women, as previously done by De Giorgi et al [21], Stenehjem et al did not confirm a significant association; however they did not stratify by menopausal status owing to the low number of postmenopausal participants at baseline [24].

The relationship between BMI and Breslow thickness has been examined in other investigations.

Fang et al enrolled 1,804 patients with melanoma from 1998 to 2008, and BMI information was available for 1,186 patients. They found that increased BMI was weakly associated with increased tumor thickness, and also with older age and increased log [CRP] [18].

In a retrospective study of 261 patients diagnosed with primary cutaneous melanoma in 7 Korean centers between 1997 and 2017, overweight and obesity statuses (BMI>23 kg/m^2^) were significantly associated with increased Breslow thickness [19]. A multivariate Cox’s proportional hazards analysis gave a HR value of 25.62 (95% CI 5.44–120.65; P < 0.001) for the association between Breslow thickness and BMI categories more than 25 kg/m^2^.

In 100 melanoma patients enrolled within days of their melanoma diagnosis in Brisbane, Australia, there was a positive association between BMI and Breslow thickness (OR 1.12, 95% CI 1.01–1.26; P = 0.04 per unit increase in BMI) [25].

In a total of 340 Italian patients with melanoma (mean Breslow thickness of 0.4 mm), a significant correlation between BMI and Breslow thickness (P < 0.01) was noted [12].

A recent analysis of the Leeds Melanoma Cohort has evidenced that BMI was independently associated with thicker melanomas [13].

There are few data regarding the association of BMI with other histological features that have a prognostic value.

Skowron et al, in their study focusing on Breslow thickness, observed that ulceration was more frequent in obese patients, although the trend was not significant [23]. Similarly, von Schuckmann et al noted that overweight/obese status was positively associated with ulcerated melanoma, but, again, not significantly [26].

The evaluation of the Leeds Melanoma Cohort showed that higher BMI was associated with ulceration, in the univariable analysis, but this association did not persist in the multivariable analysis. Ulceration was also associated with lower vitamin D levels [16].

In an Italian cohort, a significant correlation between BMI and mitotic rate (P = 0.02) was seen [12].

In another Italian study, absence of tumor infiltrating lymphocytes, a finding that appears to predispose to metastatic melanoma, was detected more frequently in obese than in non-obese melanoma patients (OR=3.92, 95% CI 1.31–11.7; P = 0.010) [27].

## Discussion

The relationship between obesity and melanoma appears to be complex.

There are conflicting findings regarding the association between obesity and risk of cutaneous melanoma [4,5]. Some cohort and case-control studies showed a variable positive correlation between obesity and melanoma risk, at least in men, while other studies found no convincing proofs of any association [5,10,28–32]. Caution should be used for the interpretation of such data. First, evidence for an association does not necessarily support a causal relationship. Moreover, methodological aspects differ between studies and could explain such divergences. It has been suggested to consider the possible effect of residual confounding by environmental and lifestyle risk factors, such as sunlight exposure, among obese and overweight subjects, as well as the variation of both BMI and other factors over time [29].

The assessment of the association between BMI and different clinical outcomes in melanoma patients (mortality/survival, risk for recurrence, sentinel node positivity, and metastases) yielded mixed results, with absence of any apparent influence according to some studies [10–16], better prognosis for overweight subjects but not for obese patients shown in one study [16], and worse outcomes for increased BMI values found by other authors [17–19]. The unfavourable outcome associations observed by Fang et al were weakened or lost their significance after adjustment for CRP, suggesting that elevated BMI can affect melanoma progression through mechanisms related to metabolic syndrome and/or chronic systemic inflammation, as indicated by CRP concentration [18].

Various reports have highlighted the link between BMI and Breslow thickness [12,13,18,19,21–25], a well-established prognostic factor of cutaneous melanoma [33].

A great part of the literature exploring the obesity-melanoma connection is based on studies that adopted BMI levels to define obesity. Nevertheless, BMI is thought to be an imperfect measure of body composition that is not able to differentiate between muscle and adipose tissues or to provide information on the distribution of adipose tissues, whether central, peripheral or in the context or proximity of target organs [2,34]. It is increasingly accepted that other parameters (eg, hip circumference, waist circumference, waist-to-height ratio and waist-to-hip ratio) may more accurately reflect body fat distribution. Furthermore, BMI can be a less accurate marker of adiposity among older people, due to the natural trends toward reduction in height, loss of muscle and increase of adipose tissue in ageing, especially in post-menopausal women [2]. Regardless of the above-mentioned limitations and the need of further studies specifically designed to overcome such limitations, BMI may be interpreted as a marker reflecting, at least partially, the effect of genetic and biological mechanisms, as well as lifestyle and environmental factors.

The biological mechanisms underlying the obesity-cancer link seem to be intricate and are still unclear. Ever-growing evidence supports the involvement of adipose tissue in tumor development and progression via endocrine and/or paracrine pathways, secreting a variety of molecules that alter systemic and local microenvironments [1]. The obesity-related dysfunctional adipose tissue and the active cross talk between adipocytes and melanoma cells can contribute to melanoma aggressiveness and progression through the release of pro-inflammatory, pro-angiogenic and lymphangiogenic factors, as well as extracellular matrix remodelling molecules, fatty acids, and probably other substances contained into the adipocyte exosomes [4,35–40].

The obesity-related changes include a chronic state of low-grade inflammation, adipokine imbalances, elevated levels of growth factors, and hormones, such as insulin, insulin-like growth factor (IGF)-1, and estrogens [1,3]. The cytokine profile of adipose tissue includes specific adipokines that may interfere with cellular processes by acting on signaling pathways, such as PI3K/Akt, MAPK, and JAK/STAT [41]. In obesity, adipocytes produce less adiponectin, that has anti-inflammatory and anti-neoplastic effects, but more leptin, that can contribute to melanoma growth and metastases [4,5,15].

Experimental findings suggest the association between obesity and aggressive tumor biology with a “meta-inflammatory” state, increased immune aging and T cell dysfunction [7].

A direct role of obesity on melanoma growth and progression has been documented by investigations in animal models. Diet-induced obesity has been demonstrated to increase melanoma progression in mice [42], while controlling obesity has proved to reverse the effect on melanoma progression [43].

The association between obesity and melanoma might be conditioned by many other factors, such as genetic mechanisms and insulin resistance, as well as the increased body surface, gut microbiota dysbiosis, and decreased levels of vitamin D [5,29,44,45].

Several data were suggestive of an inverse association between vitamin D serum levels and melanoma thickness at diagnosis [17,25,45,46]. A recent study reported that BMI and low vitamin D levels were independently associated with thicker tumors [13]. Moreover, Moreno-Arrones et al, while registering decreased vitamin D serum levels at melanoma diagnosis, described a significant association of this finding with both tumor mitotic rate and ulceration and a borderline association with Breslow thickness and BMI [47].

Genetic mechanisms have also been implicated, although the available data are still inconclusive. A genetic link between obesity and pigmentation has been proposed, as well as the role of obesity susceptibility loci in determining the risk and aggressiveness of melanoma, involving, for instance, certain vitamin D receptor polymorphisms and genetic variations in IGF-1 or estrogen receptor pathways [5,45,48,49]. A strong association between Breslow index and the *IGF-1(CA*)^19^ repeat frequency was found (P < 0.001) in one study [50]. Fang et al investigated some BMI-associated single-nucleotide polymorphisms (SNPs) in relation with melanoma risk or outcome. In particular, the C allele in the rs17782313 SNP (within the melanocortin-4 receptor) was associated with increased BMI and poorer overall and melanoma-specific survival among patients with stage I/II melanoma, showing a trend towards the association with elevated CRP [18].

Beyond biological mechanisms underlying the relationship between BMI and melanoma thickness, Stenehjem et al tried to explain their results also based on behavioural mechanisms [24]. In particular, obesity and body dissatisfaction have been associated with reduced skin self-examination and consequently with the risk of delayed detection of lesions, whereas the decline in adjusted mean Breslow thickness for the highest anthropometric values observed by those authors in their study might reflect a less sun-seeking attitude. Moreover, according to Skowron et al [23], obese people could be at higher risk of hidden melanomas because of their larger skin surface and folds or may be reluctant to undergo dermatological examinations. Nevertheless, the results of the study performed by Skowron et al did not confirm any association between BMI categorization and either the visibility of melanoma or the mode of melanoma identification [23].

## Conclusions

There are several hints suggesting the potential influence of obesity on malignancies, including melanoma. Most studies defined obesity based on BMI, although this is considered an imperfect measure of body composition. Mixed results have been obtained from studies assessing clinical outcomes in patients with melanoma in relation to BMI. More consistent data seem to support the relationship between BMI and Breslow thickness. Multiple biological and behavioural mechanisms might contribute to the effects of obesity on melanoma progression and outcome and some of these have been outlined, although the obesity-melanoma relationship deserves further investigations.

## Figures and Tables

**Table 1:** Principal Studies Exploring the Relationship Between BMI and Breslow Thickness: General Aspects and Characteristics of Melanoma Cases

Authors	De Giorgi et al [21]	Gandini et al [22]	Skowron et al [23]	Stenehjem et al [24]
Study type	Retrospective caseseries study (single center, Florence, Italy)	Hospital-based multicenter study (Italy)	Cross-sectional study in a prospective cohort (single center, Valence, France)	Prospective population-based cohort study; linkage to National Cancer Registry (Norway)
Study period	Jan 1998–Jan 2009	Dec 2010–Dec 2013	May 2007–May 2010	1972–2014
Cases of primary melanoma examined	605 86.4% thin melanomas (including *in situ* melanomas)	2738 50% thin melanomas; 29% very thick melanomas; 25% ulcerated melanomas; 1% with distant metastases; 13% with lymph node involvement	427 65% thin melanomas; 17.6% very thick melanomas	2570 53.4% thin melanomas; 23.7% very thick melanomas; 4% with regional metastases, 1% with distant metastases; 16% unspecified
Excluded cases	Patients < 40 yrs of age (when stratifying by age, because of their low number)	ALM, mucosal, *in situ* and retrospective melanomas	*In situ*, recurrent, ocular, mucosal and metastatic melanomas	Not histologically verified or retrospective melanomas
Gender	55% women; 45% men	49% women; 51% men	50% women; 50% men	44.6% women; 55.4% men
Age, yrs	Mean 53.06 (SD 16.02)	Median 55	Mean 57.74 (SD 16.1)	Mean age at diagnosis 60
Height and weight	At the first visit, measured by a physician	Collected through a self-administered questionnaire	Measured (information not further specified)	Measured by trained staff
Mean BMI (SD), kg/m^2^	24.78 (4.09)	Not reported	25.38 (4.61)	24.7 (3.3)
BMI categories, kg/m^2^ (%)	<25 (58%); ≥25 (42%)	<25 (47%); ≥25 (53%)	<25 (49%); 25–29.9 (38%); ≥30 (13%)	< 18.5; 18.5–22.9; 23–24.9; 25–27.4; 27.5–29.9; ≥30 (% not reported)
Main statistical and methodological information	Effect of BMI on the risk of thick melanoma estimated in terms of OR using a logistic regression analysis. Stratification for sex and age classes, with adjustment for age (linear) within each age class and histological subtype. Breslow thickness stratified into two groups: thick and thin	Multivariate analyses with Breslow thickness as the response variable. Multivariate random effects models, with center as a random factor; multivariate logistic models, taking into account possible confounding factors (including age, gender, educational and professional level, phenotype, residence, season of diagnosis, speciality of diagnosing doctor). Breslow thickness stratified into two groups: thin and thick, considering also very thick melanoma and additionally evaluating Breslow thickness as a continuous measure	Univariate and multivariate analyses, accounting for significant clinical and histopathological features. Breslow thickness stratified in four groups following the AJCCMS, 7^th^ Ed.	Linear regression with log_e_-transformed Breslow thickness (relationship with Breslow thickness as a continuous outcome). Adjustment for age at diagnosis, sex, ambient UV radiation of residence, average intensity of sunburns, occupational UV exposure, physical activity, education, smoking status, height. Use of adjusted mean values of Breslow thickness (in mm) by restricted cubic splines in generalized linear regression models (shape of the associations with Breslow thickness).
Site of melanoma, %	Not reported	Not reported	Trunk 48%; head and neck 15%; upper limbs 13%; lower limbs 24%	Trunk 51%; head and neck 11%; upper limbs 12.5%; lower limbs 24%; not specified 1.5%
Histopathological subtype of melanoma	SSM 89.8%; UM 1.15%; LMM 3.3%; NM 2.65%; ALM 1.8%; RM 1.3%	Not reported	SSM 62%; UM 17%; LMM 13.5%; NM 4%; ALM 3.5%	SSM 63%; NM 19%; other 4%; not specified 14%
Breslow thickness (mm)	Mean 0.87 (SD 1.07)	Not reported	Mean 1.36 mm (SD 2.47)	Median 1.0 (IQR 0.6–2)

Thin melanoma = Breslow thickness ≤1 mm; thick melanoma = Breslow thickness > 1 mm; very thick melanoma = Breslow thickness > 2 mm

AJCCMS= American Joint Committee on Cancer Melanoma Staging; ALM= acral lentiginous melanoma; BMI= body mass index; IQR= interquartile range; LMM= lentigo maligna melanoma; NM= nodular melanoma; OR= odds ratio; RM= rare melanoma; SD= standard deviation; SSM= superficial spreading melanoma; UM= unclassifiable/unclassified melanoma; UV= ultraviolet
